# Sudden hearing loss in a melanoma patient on pembrolizumab: an etiology not to be omitted in the differential diagnosis

**DOI:** 10.1186/s40425-017-0226-5

**Published:** 2017-03-21

**Authors:** Marc-Elie Nader, Jeffrey N. Myers, Paul W. Gidley

**Affiliations:** Department of Head and Neck Surgery, Unit 1445, The University of Texas MD Anderson Cancer Center, 1515 Holcombe Blvd., Houston, TX 77030 USA

**Keywords:** Immune checkpoint inhibitor, Pembrolizumab, Melanoma, Hearing loss, Leptomeningeal metastasis

## Abstract

Immune checkpoint inhibitors have emerged as a promising therapeutic option for metastatic cancers. However, they have been associated with inflammatory adverse reactions in various organ systems. A recent article reported a case of sudden bilateral hearing loss that occurred in a patient with metastatic melanoma being treated with pembrolizumab. The authors attributed that complication to an autoimmune reaction secondary to the treatment. This commentary discusses the importance of considering the diagnosis of leptomeningeal metastasis in patients with metastatic melanoma who present with new cranial nerve deficits.

## Main text

We read with great interest the paper by Zibelman and collaborators titled “Autoimmune inner ear disease in a melanoma patient treated with pembrolizumab” published in the Journal for ImmunoTherapy of Cancer [1]. The authors report a patient with widespread metastatic mucosal melanoma who developed sudden bilateral hearing loss while undergoing treatment with pembrolizumab. The episode of hearing loss was thought to be an autoimmune inner ear event secondary to the use of the immune checkpoint inhibitor.

Autoimmune inner ear disease (AIED) is defined as rapidly progressive, usually asymmetric sensorineural hearing loss [2]. Definitive tests do not exist to make this diagnosis, but a proportion of patients with AIED have antibodies against myelin P0, cochlin, β-tectorin and HSP-70 [2]. The diagnosis is often based on a careful history, physical examination, audiometry, laboratory tests and diagnostic imaging, excluding other etiologies of hearing loss.

Given that the patient had a melanoma of the anterior skull base, we respectfully point out that another etiology, namely leptomeningeal metastasis (LM), should have also been considered in the differential diagnosis for this episode of hearing loss. After carefully reading the article, it was not readily apparent if the authors had ruled out that potential cause. LM can complicate any cancer and is seen in up to 8% of all cancer patients. Melanoma is among the three most common types of malignancies that cause LM [3]. Bilateral sudden onset hearing loss has been described in the context of LM in several reports, including a patient with melanoma [4–6].

A high level of suspicion for LM must be kept in mind for patients with metastatic carcinomas who present with new cranial nerve deficits or other neurological symptoms. The diagnosis of LM may be difficult to establish. Magnetic resonance imaging (MRI) of the brain with gadolinium is the imaging study of choice. LM can sometimes be microscopic and may not show on MRI. In that case, lumbar puncture (LP) with demonstration of malignant cells in the cerebrospinal fluid can confirm the diagnosis [3]. It is unclear if the patient reported in this article underwent an MRI of the brain or an LP once hearing loss was confirmed.

It has been recently suggested that LM from melanoma may respond to systemic targeted therapy and immune checkpoint inhibitors [7]. This finding raises the possibility that the hearing loss in this patient was, in fact, caused by LM and the improvement in hearing resulted from a positive response to pembrolizumab and a decrease in disease burden. However, as the authors pointed out, there exists a potential for checkpoint inhibitors to be associated with AIED as they have been with other autoimmune related neurologic complications. It is important to remain vigilant about this possibility and to refer patients for appropriate testing and management if that pathology is suspected.

## Abbreviations

AIED: Autoimmune inner ear disease
LM: Leptomeningeal metastasis
LP: Lumbar puncture
MRI: Magnetic resonance imaging
